# Pre-existing cardiovascular diseases and glycemic control in patients with type 2 diabetes mellitus in Europe: a matched cohort study

**DOI:** 10.1186/1475-2840-9-15

**Published:** 2010-04-21

**Authors:** Alex Z Fu, Ying Qiu, Larry Radican, Donald D Yin, Panagiotis Mavros

**Affiliations:** 1 Department of Quantitative Health Sciences, Cleveland Clinic, Cleveland, Ohio, USA; 2 Global Outcomes Research, Merck & Co., Inc., Whitehouse Station, New Jersey, USA

## Abstract

**Background:**

Although there is a growing body of evidence showing that patients with type 2 diabetes mellitus (T2DM) have poor glycemic control in general, it is not clear whether T2DM patients with pre-existing cardiovascular diseases (CVD) are more or less likely to have good glycemic control than patients without pre-existing CVD. Our aim was to examine the degree of glycemic control among T2DM patients in Europe with and without pre-existing CVD.

**Methods:**

This is a matched cohort study based on a multi-center, observational study with retrospective medical chart reviews of T2DM patients in Spain, France, United Kingdom, Norway, Finland, Germany, and Poland. Included patients were aged >= 30 years at time of diagnosis of T2DM, had added a SU or a PPARγ agonist to failing metformin monotherapy (index date) and had pre-existing CVD (cases). A control cohort with T2DM without pre-existing CVD was identified using 1:1 propensity score matching. With difference-in-difference approach, logistic and linear regression analyses were applied to identify differences in glycemic control by CVD during the follow up period, after controlling for baseline demographics, clinical information, and concurrent anti-hyperglycemic medication use.

**Results:**

The percentage of case patients with adequate glycemic control relative to control patients during the 1st, 2nd, 3rd, and 4th years after the index date was 19.9 vs. 26.5, 16.8 vs. 26.5, 18.8 vs. 28.3, and 16.8 vs. 23.5 respectively. Cases were significantly less likely to have adequate glycemic control (odds ratio: 0.62; 95% confidence interval: 0.46-0.82) than controls after adjusting for baseline differences, secular trend, and other potential confounding covariates.

**Conclusions:**

T2DM patients with pre-existing CVD tended to have poorer glycemic control than those without pre-existing CVD, all other factors being equal. It suggests that clinicians may need to pay more attention to glycemic control among T2DM patients with CVD.

## Background

Patients with type 2 diabetes mellitus (T2DM) are at an increased risk of developing vascular complications. Cardiovascular diseases (CVD) are a major concern considering that the risk of cardiovascular death in patients with T2DM is double the risk of individuals without diabetes [1,2]. Patients with diabetes also have the same risk of cardiovascular death as patients with a history of myocardial infarction and no diabetes [1,3].

The literature does not seem to show a universally consistent relationship between glycemic control and CVD, despite the documented beneficial effect of glycemic control on microvascular complications [4-6]. The meta-analysis conducted by Selvin and colleagues [7] reviewed 13 prospective cohort studies, and the pooled results indicated that chronic hyperglycemia was associated with cardiovascular disease in patients with T2DM. Another meta-analysis [8] based on 8 randomized controlled trials found a similar relationship and concluded that glycemic control reduces the incidence of cardiovascular events in T2DM. A prospective epidemiological analysis based on the Heart Outcome Prevention Evaluation (HOPE) study also identified a significant relationship between glycemic level and incident cardiovascular events [9]. Most recently, Ray et al conducted a meta-analysis of five prospective randomized controlled trials and the results indicated that intensive glycemic control has cardiovascular benefits compared with standard treatment for individuals with T2DM [10].

In contrast, results from several other published studies suggest that additional research is necessary to further clarify the relationship between glycemic control and CVD [11,12]. The ACCORD (Action to Control Cardiovascular Risk in Diabetes) trial with 3.5-years follow-up found that the use of intensive therapy for glycemic control in patients with T2DM did not reduce cardiovascular events but increased mortality compared to standard therapy [13]. Meanwhile, the ADVANCE (Action in Diabetes and Vascular Disease: Preterax and Diamicron Modified Release Controlled Evaluation) trial with 5-years follow-up also did not find a significant reduction in cardiovascular events in T2DM patients with intensive treatment for glycemic control compared to patients with standard therapy [14]. Similarly, the results from another retrospective cohort study suggest that there is little or no relationship between glycemic level and recurrent cardiovascular events [11]. Clearly, it is still early to claim a definitive monotonic association between glycemic control and CVD, and additional research is warranted.

While studying this hyperglycemia-CVD relationship in T2DM, existing studies have been primarily focused on the impact of glycemic control on CVD outcomes. Few studies have addressed this relationship from a different angle. Although there is a growing body of evidence showing that patients with T2DM have poor glycemic control in general, it is not clear whether T2DM patients with pre-existing CVD are more or less likely to have good glycemic control than patients without pre-existing CVD. The purpose of the current study was to examine the degree of glycemic control among T2DM patients in Europe with and without pre-existing CVD.

## Methods

### Data source

The Real-Life Effectiveness and Care Patterns of Diabetes Management (RECAP-DM) study is a European multi-center based, epidemiological and naturalistic observational study for patients with T2DM. Using retrospective clinical chart review and patient survey at the point of visit, the RECAP-DM study was conducted in clinical practice settings in seven European countries including Spain, France, United Kingdom, Norway, Finland, Germany, and Poland. At the beginning of the study, a mailed invitation was sent to randomly selected physicians asking if they would be willing to participate in the study. The participating physicians included endocrinologists, diabetologists, general practitioners, and internalists.

**Figure 1:**
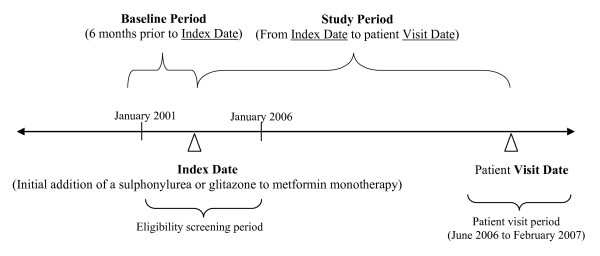
**Diagram for patient selection**.

The RECAP-DM included patients aged 30 years or older at time of diagnosis of T2DM, who had added a SU or PPARγ agonist (glitazones) to failing metformin monotherapy on a date from January 2001 to January 2006, which was defined as the index date (Figure 1). Patient enrollment in the RECAP-DM occurred during regular visits within the period from June 2006 to February 2007. Eligible patients were required to have at least one hemoglobin A1c (HbA_1c_) measurement in the 12-months prior to the visit date. Patients were excluded from the RECAP-DM if they had type 1 diabetes, were pregnant women with gestational diabetes, or had diabetes secondary to other factors (such as malnutrition, infection, and surgery). Patients who were unable to complete the questionnaires or were participating in other clinical studies were excluded as well. To our knowledge, RECAP-DM is the only study applying a consistent methodology across multiple European countries focusing on patients with T2DM who also received combination oral diabetes medications [15,16].

### Study Design and Variables

The current study was a matched cohort study using the RECAP-DM sample. The case cohort included those who had pre-existing CVD (i.e., with onset date prior to the index date) within the RECAP-DM and the control cohort was selected among those who had no pre-existing CVD. CVD included ischemic heart disease, myocardial infarction (MI), stroke, and peripheral vascular disease based on ICD-9 codes. Patient baseline information was collected and included patient demographic characteristics and clinical information during the 6-months prior to the index date.

The primary outcome of focus was HbA_1c _at baseline as well as during the study period (from index date to patient visit date - Figure 1). Another outcome of interest was the proportion of patients with adequate glycemic control, defined as HbA_1c _< 6.5% by the 2005 International Diabetes Federation [17]. To evaluate the pattern of glycemic control over time, patients were grouped based on time, categorized in years from the index date to the visit date. For each year, only samples with HbA_1c _measures within the year were included. If there were multiple HbA_1c _measures within the year for a patient, the most recent measurement relative to the visit date was selected.

Other baseline covariates that were controlled for in the analysis included age, gender, ethnicity, duration of T2DM, alcohol consumption status, physical activity frequency, country location, body mass index, physician specialty, and duration of metformin use. The participating physicians included endocrinologists, diabetologists, general practitioners, and internalists for RECAP-DM. Endocrinologists or diabetologists may be more acutely aware of disease management needs relative to general practitioners or internalists, which could influence the glycemic outcomes of their patients. Thus, a classification of endocrinologists/diabetologists versus general practitioners/internalists was applied for physician specialty. The index medication use of SU versus glitazones was controlled for as well.

During the study period, patients may not be persistent in their use of the index medication. The treatment pattern could change, which would confound the evaluation of the relationship between baseline CVD and the HbA_1c _at various time. Therefore, the concurrent anti-hyperglycemic medication treatment at the time of the HbA_1c _measurement was also captured and controlled for in the analysis. If the date of the HbA_1c _measurement was between the starting and stop dates of a certain anti-hyperglycemic medication, that medication was defined as the concurrent treatment at the time of that HbA_1c _measurement. In the analyses, the concurrent treatment was categorized into 6 groups: metformin+SU combination therapy, metformin+glitazones combination therapy, SU monotherapy, glitazones monotherapy, metformin monotherapy, and therapies with insulin. There might be slight overlap between certain treatment types, such as the combination therapy and therapies with insulin. Thus, the estimated effect on any treatment type reflected the marginal difference between patients with that therapy and those without any of the 6 aforementioned treatments.

### Statistical Analyses

Descriptive analysis was conducted to summarize patient demographic characteristics and clinical information at baseline. The variables were compared between patients with and without CVD at baseline. T-tests were used for continuous variables and chi-square tests were calculated for categorical variables.

The propensity score method was used to match case and control cohorts. A logistic regression was first used to predict the probabilities of pre-existing CVD using a list of baseline characteristics. The variable selection was based on the idea [18,19] that it was the variables having an effect on or associated with the HbA_1c _and blood glucose instead of CVD that needed to be matched so that the impact of pre-existing CVD on HbA_1c _can be properly evaluated. The variables that did not have an effect on HbA_1c _outcomes were not included.

The case and control cohorts were 1:1 matched by propensity scores using the greedy matching algorithm [20]. That is, once a control is matched, the control is not reconsidered. The algorithm makes "best" matches first and "next-best" matches next, in a hierarchical sequence until no more matches can be made. Best matches are those with the highest digit match on propensity score. First, controls were matched to cases on 8 digits of the propensity score. For those that did not match, controls were then matched to cases on 7 digits of the propensity score. The algorithm proceeds sequentially to the lowest digit match on propensity score (1 digit).

The trends of glycemic control and the impact of pre-existing CVD on glycemic control were analyzed using a difference-in-difference (DID) strategy [21,22]. The DID approach compares the pre- and post-index difference in glycemic control among T2DM patients with pre-existing CVD, with the pre- and post-index difference in glycemic control among T2DM patients without pre-existing CVD. Thus, the DID strategy allows one to identify the effect attributable to pre-existing CVD after accounting for any possible secular trend.

Logistic and linear regression analyses were applied to assess any relationship between pre-existing CVD and glycemic control (HbA_1c_<6.5% yes/no) or HbA_1c _value. For the DID approach, 5 additional binary variables were included in the regression analyses indicating the 5 post-index time periods (<1 year, 1-2 years, 2-3 years, 3-4 years, and ≥4 years from the index date). These were used to capture the time effect on outcomes. Five interaction terms for CVD with the 5 post-index periods were included in the model, capturing the potential differential CVD-effects on glycemic control over time.

As a sensitivity analysis, the time effect was assumed linear after the index date. Thus, the analytical model was reduced where only 2 time variables were included, one binary variable indicating the time of post versus pre-index and another continuous variable representing the actual time from the index date measured in years. Subsequently, for the sensitivity analysis, 2 interaction terms were included to capture the potential differential CVD-effects on glycemic control. Robust variance estimator was used to account for multiple observations per patient and correlation within subjects.

In our study, several covariates had missing or unknown data for certain patients. Although the missing for each covariate is trivial, the final sample size would have been reduced to less than half of the total if the complete case analysis approach was adopted. We used the multiple imputation procedure to impute missing values of each covariate, assuming that the data are multivariate normally distributed and the missing data are missing at random. The procedure used the Markov Chain Monte Carlo method with a single chain to create imputations.

## Results

This study had 1942 T2DM patients with complete questionnaire information for CVD at the baseline. There were 406 patients (20.9%) with CVD and 1536 patients without CVD before the index date. Among those with pre-existing CVD, ischemic heart disease was the most prevalent (272 patients, 67%), followed by MI (103 patients, 25%), peripheral vascular disease (98 patients, 24%), and stroke (47 patients, 12%).

The descriptive comparisons of all the demographics and baseline clinical information between patients with and without pre-existing CVD are shown in Table 1. Compared to patients without CVD at the baseline, those with CVD were significantly older and more likely to be male. UK and Poland had more patients whereas Spain and Finland had fewer patients with pre-existing CVD in the sample. The percentage of the sample with adequate glycemic control (HbA_1c_<6.5%) was significantly lower for those with pre-existing CVD compared to those without. There were significantly more patients with pre-existing CVD who added SU instead of glitazones to metformin monotherapy at the index date compared to patients without CVD.

**Table 1:** Patients characteristics by cardiovascular diseases at baseline (before and after propensity score 1:1 matching)

	Before Matching			After Matching		
	
Variables	Mean (S.D.)		P-value	Mean (S.D.)		P-value
				
	With CVD (N = 406)	Without CVD (N = 1536)		With CVD (N = 394)	Without CVD (N = 394)	
SU added at index	80.8%	73.6%	0.003	-	-	-

Glitazones added at index	19.2%	26.4%		-	-	-

**Demographics**						

Age (years)	64.3 (9.2)	59.5 (10.5)	< 0.0001	64.0 (9.0)	63.9 (10.1)	0.96

Male	65.5%	51.3%	< 0.0001	65.0%	70.3%	0.11

Caucasian	98.0%	96.7%	0.17	98.0%	98.4%	0.68

Duration of diabetes (years)	6.1 (5.2)	5.6 (4.8)	0.09	6.0 (5.0)	6.1 (5.0)	0.99

Never used alcohol	27.6%	30.4%	0.29	27.9%	26.1%	0.57

Physical Activity						

No regular activity	39.1%	34.6%	0.11	37.6%	39.5%	0.58

< 3 times/week	39.1%	39.7%	0.83	41.0%	36.5%	0.12

≥ 3 times/week	21.9%	25.7%	0.12	21.4%	24.0%	0.34

Country						

Spain	15.8%	28.1%	< 0.0001	16.2%	15.0%	0.62

France	6.9%	8.1%	0.43	7.1%	6.9%	0.89

UK	26.6%	16.9%	< 0.0001	26.7%	26.4%	0.94

Norway	3.0%	3.4%	0.67	3.1%	4.8%	0.20

Finland	7.4%	10.7%	0.049	7.6%	10.2%	0.21

Germany	17.7%	21.4%	0.10	18.3%	19.8%	0.59

Poland	22.7%	11.5%	< 0.0001	21.1%	17.0%	0.15

**Baseline Clinical Information**						

HbA1c (%)	8.1 (1.3)	8.0 (1.4)	0.69	8.06 (1.26)	8.05 (1.39)	0.96

With HbA1c < 6.5%	5.2%	8.3%	0.037	5.1%	7.6%	0.14

Body mass index (kg/m^2^)	31.7 (8.2)	31.9 (6.2)	0.60	31.7 (6.6)	31.7 (6.7)	0.94

Physician specialty						

Endocrinologists/Diabetologists	40.6%	40.6%	0.99	40.6%	41.1%	0.89

General practitioners/Internists	59.4%	59.4%		59.4%	58.9%	

Duration of metformin use (years)	3.0 (2.9)	2.8 (2.5)	0.15	2.9 (2.7)	2.8 (2.5)	0.60

Note: CVD: cardiovascular diseases; S.D.: standard deviation.

Patient characteristics after propensity score 1:1 matching are also listed in Table 1, where the created case and control cohorts were comparable regarding baseline characteristics. Of the 394 patients with pre-existing CVD, 65% were male, mean (standard deviation) age and duration of T2DM was 64.0 (9.0) and 6.0 (5.0) years, respectively.

**Figure 2:**
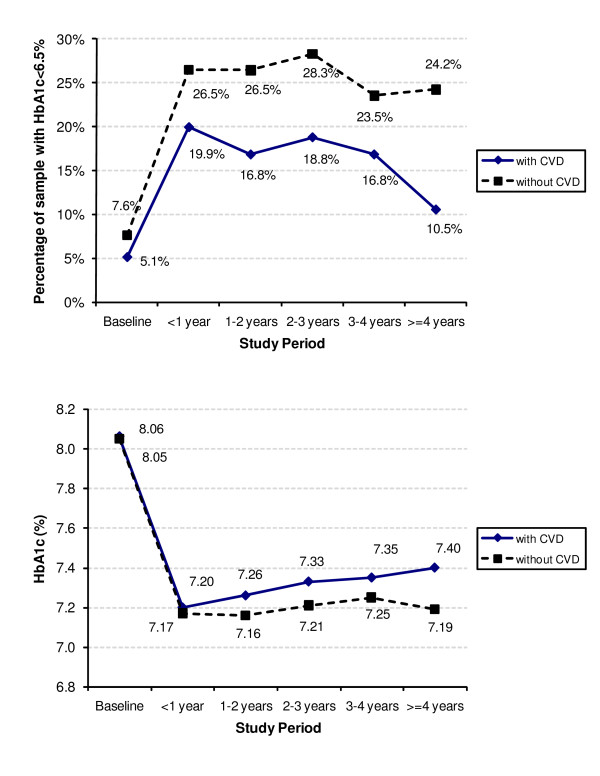
**Glycemic control over time**.

Figure 2 shows the percentage of the sample with HbA_1c_<6.5% (adequate glycemic control) and the average HbA_1c _over time. The percentage of the case cohort with adequate glycemic control relative to the control cohort during the 1st, 2nd, 3rd, and 4th years after index date was 19.9 vs. 26.5, 16.8 vs. 26.5, 18.8 vs. 28.3, and 16.8 vs. 23.5 respectively. The sample sizes remained almost identical between the case and control cohorts although both decreased over time owing to attrition.

None of the interaction terms between pre-existing CVD and the post-index time periods in the regression analyses were statistically significant, indicating no differential CVD-effects on glycemic control over time. Thus, the final regression models excluded these interaction terms. The results from the final regressions are reported in Table 2 and indicate that patients with pre-existing CVD were significantly less likely to have adequate glycemic control (odds ratio: 0.62; 95% confidence interval: 0.46-0.82) than those without pre-existing CVD after controlling for other potential confounding covariates. The effect was not significant when HbA_1c _value was the outcome of focus. The almost identical results for CVD between regressions on the same outcome variable implies that treating the post-index time continuously or categorically year-by-year had little difference on the coefficient of interest. All time covariates were significant, indicating that the likelihood of adequate glycemic control was higher for post-index outcomes compared to their pre-index values. This demonstrates the effectiveness of the post-index medication treatment regardless of the patient cohorts. Such a finding is important for the original RECAP-DM study.

**Table 2:** Impact of pre-existing cardiovascular diseases on adequate glycemic control

*Logistic regressions on percentage of patients with HbA_1c _< 6.5%*			
**Variable**	**Odds Ratio**	**95% confidence intervals**	**P-value**

With CVD vs. without CVD	0.62	0.46, 0.82	0.001

Time indicators			

<1 year from index	6.21	3.61, 10.70	<0.001

1-2 years from index	6.39	3.72, 10.98	<0.001

2-3 years from index	6.69	3.81, 11.75	<0.001

3-4 years from index	7.66	4.24, 13.87	<0.001

>= 4 years from index	5.62	3.02, 10.46	<0.001

			

**Variable**	**Odds Ratio**	**95% confidence intervals**	**P-value**

With CVD vs. without CVD	0.62	0.46, 0.82	0.001

Study period vs. baseline	6.47	3.71, 11.29	<0.001

Years after the index	1.00	0.92, 1.10	0.96



*Linear regressions on HbA_1c _(%)*			

**Variable**	**Coefficient**	**95% confidence intervals**	**P-value**

With CVD vs. without CVD	0.02	-0.10, 0.14	0.75

Time indicators			

<1 year from index	-0.80	-0.97, -0.62	<0.001

1-2 years from index	-0.78	-0.96, -0.60	<0.001

2-3 years from index	-0.76	-0.96, -0.57	<0.001

3-4 years from index	-0.88	-1.07, -0.68	<0.001

>= 4 years from index	-0.84	-1.07, -0.60	<0.001

			

**Variable**	**Coefficient**	**95% confidence intervals**	**P-value**

With CVD vs. without CVD	0.02	-0.10, 0.14	0.75

Study period vs. baseline	-0.78	-0.97, -0.59	<0.001

Years after the index	-0.01	-0.05, 0.03	0.65

Note: CVD: cardiovascular diseases. Other controlled covariates in the regression analyses: concurrent treatment of the HbA_1c _measurement (metformin+SU combination therapy, metformin+glitazones combination therapy, SU monotherapy, glitazones monotherapy, metformin monotherapy, any therapies with insulin), index SU vs. glitazones, age, gender, Caucasian, duration of diabetes, never used alcohol, physical activity, country, BMI, physician specialty.

## Discussion

Our study results suggest that there is a significant difference in the percentage of T2DM patients with adequate glycemic control (HbA_1c _< 6.5%) in those with and without pre-existing CVD. Using a seven-country European sample, the current study provides important empirical evidence about the degree of glycemic control among T2DM patients with and without pre-existing CVD. Most of the previous research has focused on CVD resulting from poor glycemic control as the outcome of interest. The present study is unique in that we used HbA_1c _value and adequate glycemic control as the outcomes of interest and studied CVD as a baseline factor. Due to the design of the RECAP-DM study, the temporal relationship between CVD and glycemic control is apparent.

The premise of the study is that, other things being equal, patients with pre-existing CVD should have better glycemic control due to increased risk of future cardiovascular events. Our study showed that patients at increased risk of cardiovascular events are not controlled any better than patients with lower risk (and in fact their control is worse). This implies an important unmet medical need. The reasons for the observed difference in glycemic controls between the two groups remain a question. We have adjusted for the concurrent anti-hyperglycemic medication use in the analyses. Thus, the treatment difference might not be a contributor. However, the literature indicates that physicians taking care of diabetic patients with CVD might face multiple obstacles for obtaining adequate glycemic control. Treatment guidelines suggest more stringent control of blood pressure and lipids, in addition to the blood glucose control. There are a variety of additional medications (e.g., antiplatelet medications) that are recommended to prevent future CVD events. More importantly, there is evidence that glycemic control may not be the most significant factor for preventing another CVD event [23,24]. Diabetic patients with CVD may have conditions (e.g., congestive heart failure) that prevent the use of certain anti-hyperglycemic agents. Additionally, due to concomitant conditions and medication interactions, these patients may be at particularly high risk for hypoglycemia, which may cause even more morbidity than mild hyperglycemia in the short term. Obviously, more research is needed in this area.

Our analyses have several strengths. First, this study used the RECAP-DM sample, which was recruited using a consistent methodology across seven European countries focusing on T2DM patients who received combination oral diabetes treatment. Second, we applied the DID strategy in the analysis design. This approach cancelled out both the secular trend and the baseline group difference while evaluating HbA_1c _differences between patients with and without pre-existing CVD in the follow-up period. Further, the DID method assumes the comparison groups exhibit the same trend over time as the null hypothesis. This assumption holds better when the baseline difference is small [22]. This is the case for the current study where no significant difference was observed for both HbA_1c _value and the percentage of patients with adequate glycemic control for the matched samples.

In this study of European patients with T2DM, the majority of patients had not reached the goal of adequate glycemic control with HbA_1c _< 6.5%. This might partially be explained by the nature of the RECAP-DM sample which comprised patients who failed metformin monotherapy. Existing studies based on RECAP-DM [15] also showed that more and more patients used therapies with insulin over time, which indicated intensification of the medication treatment for this group of T2DM patients. It is also likely that most of the patients in this sample had moderate to severe T2DM. As indicated by our study results, the proportions of patients with adequate glycemic control decreased over time. This reflected the progressively deteriorating nature of T2DM, which has been demonstrated in the literature [25,26]. Unfortunately, due to lack of data we did not include analyses on dosage information on the anti-hyperglycemic agents used after the index date. This could have provided useful information regarding differences in intensification regimens between case and control cohorts. Nonetheless, the suboptimal glycemic control seen in CVD patients could also have resulted from physicians' fear of increased mortality risk, which was observed in the ACCORD trial [13]. Additionally, using the interaction terms in the regression analysis, we planned to test whether or not the HbA_1c _of patients with CVD tended to deteriorate more quickly than the HbA_1c _of patients without CVD. The results indicated that the differential effect among our sample was not statistically significant.

Due to the 1:1 propensity score matching, the final sample (N = 788) was considerably reduced from the original (N = 1942). A sensitivity analysis with propensity score 1:3 matching with replacement was conducted (N = 1620) and the results were similar. We decided to use the 1:1 matching as our primary analysis because of the greater internal validity of the design. Further, an alternative approach using last observation carried forward was adopted as a sensitivity analysis, and similar results were found. It is also worth noting that we applied a multiple imputation procedure to impute the missing values of each covariate. Sensitivity analysis excluding patients with missing data was conducted (complete case analysis) and as expected, the regression results were numerically similar but non-significant, which was likely due to the smaller sample size (N = 430).

Although a noteworthy difference in adequate glycemic control was identified between T2DM patients with and without pre-existing CVD, this study has several limitations. First, this group of selected patients all had SU or PPARγ agonist added to metformin monotherapy, and they were recruited through their physicians who had agreed to participate in the RECAP-DM study. These physicians may be more motivated due to their willingness to participate and their patients may not represent the overall population of patients with T2DM in Europe. Second, the CVD status was collected at baseline. Patients without CVD before the index date may develop CVD over time, which leads to potential misclassification of the CVD status. Nevertheless, this effect can only lessen the potential difference to be identified. With a significant finding at present, the true difference in glycemic control in those with and without pre-existing CVD would have been larger if the potential misclassifications were to be considered. Third, the biochemical marker of microalbuminuria was not collected in the baseline period. Given that T2DM patients are at significantly increased risk of cardiovascular events [27], it would have been important to control for other key risk factors for CVD such as microalbuminuria. Fourth, as is typical with any observational study, there may have been other unobserved confounding factors not available in the data (e.g., other comorbid conditions) that could have led to residual confounding.

## Conclusions

Based on a seven-country European sample, we found that T2DM patients with pre-existing CVD tended to have poorer glycemic control than those without pre-existing CVD, all other factors being equal. This implies a need for building awareness, education, and novel effective (or more aggressive) treatments for T2DM patients with CVD. Current treatments may not be adequate in this population. It is widely recognized that achieving specific glycemic goals in patients with diabetes can substantially reduce diabetes-related complications. Since patients with pre-existing CVD have a higher risk of future diabetes-related complications, clinicians may want to pay more attention to glycemic control in these high risk patients.

## Competing interests

AZF received research grant support from Merck & Co., Inc. for this study. YQ, LR, DDY, and PM are employed by and are share holders of Merck & Co., Inc.

## Authors' contributions

All authors contributed to the design of this study. AZF and PM performed the statistical analyses. All authors contributed to the interpretation of the study results and writing process, and approved the final manuscript.
